# Mitochondrial respiration and dynamics of *in vivo* neural stem cells

**DOI:** 10.1242/dev.200870

**Published:** 2022-11-30

**Authors:** Stavroula Petridi, Dnyanesh Dubal, Richa Rikhy, Jelle van den Ameele

**Affiliations:** ^1^ Department of Clinical Neurosciences and MRC Mitochondrial Biology Unit, University of Cambridge, Cambridge CB2 0XY, UK; ^2^ Biology, Indian Institute of Science Education and Research, Homi Bhabha Road, Pashan, Pune 411008, India

**Keywords:** Neural stem cell, Mitochondria, Oxidative phosphorylation, Mitochondrial morphology, Reactive oxygen species, Notch

## Abstract

Neural stem cells (NSCs) in the developing and adult brain undergo many different transitions, tightly regulated by extrinsic and intrinsic factors. While the role of signalling pathways and transcription factors is well established, recent evidence has also highlighted mitochondria as central players in NSC behaviour and fate decisions. Many aspects of cellular metabolism and mitochondrial biology change during NSC transitions, interact with signalling pathways and affect the activity of chromatin-modifying enzymes. In this Spotlight, we explore recent *in vivo* findings, primarily from *Drosophila* and mammalian model systems, about the role that mitochondrial respiration and morphology play in NSC development and function.

## Teaser

**Summary:** This Spotlight explores recent findings from *Drosophila* and vertebrates on how mitochondrial respiration and morphology interact with neural stem cell transitions in the developing and adult brain *in vivo*.

## Introduction

**Fig. 1.**
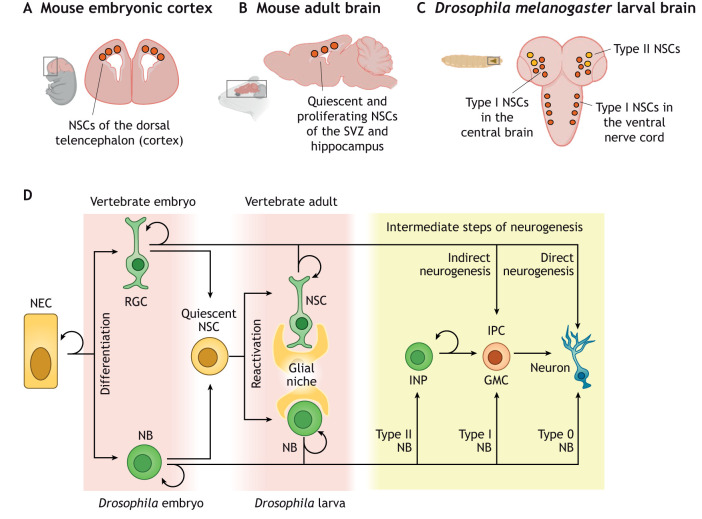
**NSC differentiation in vertebrates and *Drosophila.*** (A) Schematic of the mouse embryonic cortex showing neural stem cells (NSCs) in the dorsal telencephalon. (B) Schematic of mouse adult brain showing the presence of quiescent and proliferative NSCs in the subventricular zone (SVZ) and hippocampus. (C) Schematic of the *Drosophila* larval brain showing the presence of type I and type II NSCs in the central brain and ventral nerve cord. (D) Overview of neurogenesis in vertebrates (top) and *Drosophila* (bottom) at different developmental stages. GMC, ganglion mother cell; INP, intermediate neural progenitor; IPC, intermediate progenitor cell; NB, neuroblast; NEC, neuroepithelial cell; NSC, neural stem cell; RGC, radial glial cell.

Neural stem cells (NSCs) of the developing and adult brain (Fig. 1A-C) are a diverse population of progenitor cells that balance self-renewal with differentiation into post-mitotic neurons and glia. To generate the right number and type of progeny in the right time and space, NSCs will change their division mode and competence through a range of transitions that are conserved in many organisms (Homem and Knoblich, 2012; Okano and Temple, 2009).

A first transition occurs early in development, when NSCs in the neuroepithelium (neuroepithelial cells, NECs) switch from symmetric amplifying divisions to asymmetric divisions that allow self-renewal while also generating more differentiated progeny (Fig. 1D). Asymmetric dividing NSCs are called neuroblasts (NBs) in *Drosophila* and radial glial cells (RGCs) or NSCs in the vertebrate brain. Depending on the context and organism, these NSCs reside in very different environments and niches. For example, larval NBs in *Drosophila* and NSCs in the adult mouse brain interact closely with nearby glia or astrocytes, whereas these glial cells are not present in the embryonic *Drosophila* or mouse brain (Fig. 1). After cell division, differentiation into postmitotic cells may occur either directly or indirectly via transit-amplifying cells, often called intermediate-progenitor cells (IPCs) or ganglion mother cells (GMCs) in *Drosophila* (Fig. 1D). Throughout development, NSCs will undergo many other transitions: they change their competence over time in a process called temporal patterning; they take on different identities in response to various spatial cues; after embryogenesis, they may cease proliferation temporarily to undergo periods of quiescence (Fig. 1D); and, finally, they will disappear through terminal differentiation or apoptosis.

Over past decades, much progress has been made in our understanding of the key signalling pathways and transcription factors that regulate these NSC transitions (Taverna et al., 2014; Tiberi et al., 2012a). However, all these transitions occur in the constantly changing environment of the developing or adult brain and are accompanied by profound changes in cell morphology, cell behaviour, membrane composition and chromatin structure. How cellular metabolism adapts to meet the very specific and diverse metabolic requirements of these states and environments, and whether rewiring of metabolic pathways may not only accompany, but also drive, these changes in NSC behaviour is only now beginning to emerge (Mosteiro et al., 2021).

Mitochondria, which are commonly known as the powerhouses of eukaryotic cells, have emerged as central players in metabolic reprogramming to determine NSC fate. They are highly dynamic organelles with a double membrane and their own circular genome: the mitochondrial DNA (mtDNA). Mitochondrial oxidative phosphorylation (OxPhos) is a major contributor to ATP production (Box 1), but mitochondria are also involved in a wide range of other processes. By regulating intracellular messaging molecules, such as calcium (Ca^2+^) and reactive oxygen species (ROS) (Box 2), and controlling the availability of metabolites, such as acetyl-CoA, α-ketoglutarate or NAD^+^ that are required for post-translational modifications of signalling proteins and histones, one can easily envisage a crucial role for mitochondrial metabolism in NSC transitions.

**Box 1. Glycolysis and oxidative phosphorylation**

The two main pathways for energy production within cells are glycolysis, which primarily takes place in the cytosol, and oxidative phosphorylation (OxPhos), in mitochondria. Glycolysis metabolises glucose to pyruvate, generating two molecules of ATP per molecule of glucose. In the presence of oxygen (O_2_), pyruvate mostly enters mitochondria, where it fuels the tricarboxylic acid (TCA) cycle and OxPhos to produce 36 molecules of ATP. Pyruvate can also be converted to lactate in a process known as fermentation that is not dependent on O_2_. In hypoxia, glucose fermentation is referred to as anaerobic glycolysis; aerobic glycolysis occurs when pyruvate is preferentially converted to lactate despite the presence of O_2_, which happens under specific conditions, e.g. in some proliferating cell types (Vander Heiden et al., 2009).

OxPhos consists of the electron transport chain (ETC), composed of four enzyme complexes (I-IV) within the inner mitochondrial membrane, where electrons pass through a series of redox reactions (dashed line in the figure) to release energy. Three complexes (I, III and IV, also known as NADH dehydrogenase, cytochrome C reductase and cytochrome C oxidase, respectively) use the energy to transfer protons from the mitochondrial matrix to the intermembrane space. Complex IV transfers the electrons to O_2_ as the final electron acceptor. Complex II (or succinate dehydrogenase) does not pump protons but is also part of the TCA cycle (not shown). The resulting proton gradient across the inner mitochondrial membrane is finally used by complex V (also known as ATP synthase) for production of ATP from ADP.

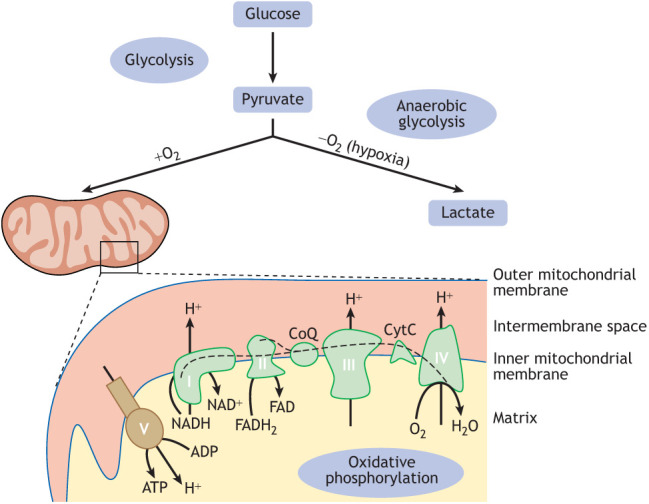

**Box 2. Reactive oxygen species in NSCs and their niche**

Although mitochondrial respiration is of vital importance to cellular homeostasis, it is also a major source of reactive oxygen species (ROS) (Murphy, 2009). ROS are often considered to be toxic by-products, but they perform key signalling functions in development and disease (Chandel, 2015; Oswald et al., 2018). In cultured mouse neural stem cells (NSCs) derived from embryonic cerebral cortex neurospheres, spontaneous bursts of ROS production have been observed that decrease NSC proliferation (Hou et al., 2012). A null mutant of superoxide dismutase 2 (*Sod2*^−/−^) exhibits increased ROS exposure, as well as significant loss of NSCs and differentiated neurons in mouse embryonic cerebral cortex (Hou et al., 2012). Forced mitochondrial fission by depletion of Opa1 or mitofusins in dissociated mouse NSCs also results in increased ROS, which in turn suppresses Notch signalling (Box 3) through elevated NRF2 activity, eventually resulting in loss of stemness and NSC depletion in the mouse brain (Khacho et al., 2016).

Various mechanisms have evolved to protect NSCs from harmful ROS, e.g. when organisms are exposed to hypoxia or oxidative stress. Lipid droplets in glial cells of the *Drosophila* NSC niche have recently attracted attention as a way to protect polyunsaturated fatty acids from harmful peroxidation (Bailey et al., 2015). Glial-specific knockdown of the lipid droplet storage regulator Lsd-2 under ROS-inducing conditions results in increased peroxidation of fatty acids and proteins in NSCs, further decreasing their proliferation (Bailey et al., 2015). It is likely that lipid droplets have effects beyond lipid homeostasis and ROS, e.g. by regulating Hedgehog pathway activity in the *Drosophila* NSC niche (Dong et al., 2021).

Much of our current knowledge regarding the role of mitochondria in NSCs and their progeny is based on detailed studies *in vitro*, in primary cultures or from pluripotent stem cell (PSC)-derived NSCs (reviewed by Iwata and Vanderhaeghen, 2021; Knobloch and Jessberger, 2017; Namba et al., 2021). However, normally, NSCs reside in a specialised microenvironment that provides cell-cell interactions, signalling cues and nutrients that together help to maintain the balance between proliferation and differentiation (Bjornsson et al., 2015). *In vivo* studies are, therefore, of particular importance, complementing these *in vitro* findings. In this Spotlight, we discuss recent findings that highlight the role of mitochondrial OxPhos and morphology in normal NSC development, and primarily focus on studies performed *in vivo* in mammalian and *Drosophila* model organisms.

## OxPhos or glycolysis: not a simple choice

The two main metabolic pathways for energy production within the cell are glycolysis, which takes place in the cytosol, and mitochondrial OxPhos (Box 1). Early observations made by Otto Warburg (Vander Heiden et al., 2009; Warburg, 1956) were the first indication that highly proliferative tumour cells in culture are less dependent on OxPhos than the differentiated postmitotic cells in the tissue from which they are derived, possibly as a normal adaptation to the metabolic requirements of proliferation (Birsoy et al., 2015; Sullivan et al., 2015; Titov et al., 2016; Vander Heiden et al., 2009). Many studies *in vivo* and *in vitro* have since shed a more nuanced light on this, and metabolic flux in proliferating stem cells or cancer cells is influenced by extrinsic and intrinsic factors, such as substrate availability, signalling pathways, tissue identity and oncogenic mutations. In the developing brain, early studies in rat or human fetuses and neonates found that overall mitochondrial mass or oxygen consumption rate (OCR) increases with gestational age (Cordeau-Lossouarn et al., 1991; Himwich et al., 1959; Yoxall and Weindling, 1998), suggesting that NSCs have fewer mitochondria and/or less OxPhos activity than their neuronal progeny. The work by Agathocleous and colleagues was seminal in this respect, because it was the first to demonstrate *in vivo* cell type-specific differences in glycolysis and mitochondrial respiration between NSCs and their progeny (Agathocleous et al., 2012). Using the *Xenopus* and zebrafish retina as a model, they showed a higher dependence on OxPhos for ATP production in differentiated retinal cells than the NSCs from which they are derived. NSCs instead rely on glycogen to produce ATP and metabolites through glycolysis, independent of tissue oxygen levels (Agathocleous et al., 2012). Since then, extensive *in vivo* studies have further elucidated the relative contributions of OxPhos and glycolysis during NSC proliferation and differentiation, mainly using the mouse and *Drosophila* brain as model systems.

### OxPhos in *Drosophila* NSCs: fuelling proliferation

**Fig. 2.**
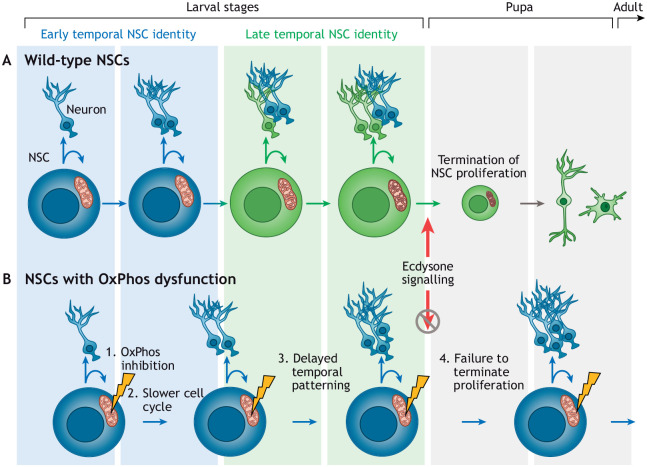
***Drosophila* NSC proliferation and differentiation.** (A) Schematic of *Drosophila* neural stem cell (NSC) self-renewal, differentiation, temporal patterning and termination of proliferation under wild-type conditions. NSCs with early temporal identity are in blue and with late temporal identity are in green. In wild-type conditions, at the transition between larval and pupal stages, a systemic pulse of the steroid hormone Ecdysone induces termination of NSC proliferation. (B) Oxidative phosphorylation (OxPhos) dysfunction in NSCs results in the slowing of the cell cycle and subsequent delayed temporal patterning. If NSCs do not transition from early to late temporal identity, they cannot respond to Ecdysone and do not undergo termination of proliferation.

NSCs in *Drosophila* are present during embryonic and larval stages (Fig. 1C,D), but not in adult flies. At the end of larval development, NSCs undergo apoptosis or symmetric division into two postmitotic cells to terminate proliferation (Homem et al., 2014; Truman and Bate, 1988) (Fig. 2A). An elegantly designed genome-wide RNAi screen for genes required for the termination of NSC proliferation showed that NSC-specific OxPhos inhibition causes NSCs to persist in the adult *Drosophila* brain (Homem et al., 2014). This observation led to a widely accepted model in which OxPhos is dispensable in proliferating larval NSCs, which, instead, rely on aerobic glycolysis for ATP production and biosynthesis. At the end of larval development, in response to a systemic peak of the steroid hormone ecdysone, NSCs were thought to activate OxPhos, which causes them to shrink and undergo timely termination of proliferation (Homem et al., 2014). Several findings support the view that *Drosophila* larval NSCs do not require OxPhos for normal proliferation: NSC mitochondria are smaller than those in their differentiated progeny (Sen et al., 2013), and mutations in *Drosophila qless* (a prenyl transferase involved in synthesis of the essential OxPhos component coenzyme Q) lead to neuronal death but do not affect NSC proliferation (Grant et al., 2010). In addition, whole-organism metabolic analyses have found aerobic glycolysis as the preferential pathway to support growth during larval development (Tennessen et al., 2011).

Conversely, many observations have indicated substantial mitochondrial OxPhos activity in *Drosophila* NSCs. For example, larval NSCs respond to hypoxia by significantly increasing ROS production (Bailey et al., 2015), indicating at least the presence of a functional electron transport chain (ETC) (Murphy, 2009) (Boxes 1 and 2). This response is accompanied by increased lactate production, meaning that larval brains shift away from OxPhos towards glycolysis upon oxygen deprivation (Gándara et al., 2019). In addition, the lethality caused by a whole-body mtDNA mutation that impairs complex IV function is phenocopied by induction of this mtDNA mutation in only NSCs (Chen et al., 2015). These studies led to a more comprehensive analysis of NSC transitions in *Drosophila* larvae, showing that NSC-specific inhibition of OxPhos, but not glycolysis, affects many aspects of NSC behaviour (Van Den Ameele and Brand, 2019). Indeed, RNAi-mediated knockdown of OxPhos subunits specifically in NSCs prevents timely termination of proliferation at the end of larval development, as described previously (Homem et al., 2014). However, we also observe that OxPhos knockdown decreases the NSC proliferation rate by slowing the cell cycle at the G_1_/S transition. Slowing the cell cycle independently of OxPhos inhibition; for example, through ectopic activation of the G_1_/S (but not the G_2_/M) checkpoint (Van Den Ameele and Brand, 2019) or through fibroblast growth factor (FGF) and Hedgehog (Hh) signalling pathway activation (Dong et al., 2021), can also prevent termination of proliferation. Interestingly, prolonged G_1_/S transition leads to a striking delay in temporal patterning: NSCs with OxPhos inhibition continue to express markers and generate the types of neurons compatible with an early developmental identity, even at later developmental stages (Fig. 2B). Conversely, when temporal identity is genetically restored in NSCs with OxPhos dysfunction, timely termination of proliferation is partially rescued, in spite of continued OxPhos inhibition. It has previously been shown that prolonged expression of early temporal markers can make NSCs unresponsive to developmental cues, such as ecdysone, that govern termination of proliferation (Maurange et al., 2008; Yang et al., 2017). Therefore, in contrast to what has previously been proposed (Homem et al., 2014), it seems that the persistence of ectopic NSCs in the adult brain upon OxPhos inhibition is an important, but likely secondary, consequence of a primary phenotype during *Drosophila* larval brain development, where OxPhos, but not glycolysis, is required to drive NSC proliferation and temporal patterning.

Some of the apparent discrepancies between these observations may be explained by the different metabolic requirements of various NSCs, depending on their identity or the tissue-wide context. Although some studies (Bailey et al., 2015; Van Den Ameele and Brand, 2019) have primarily analysed the impact of OxPhos inhibition or hypoxia on ‘type I’ NSCs in the *Drosophila* ventral nerve cord (Fig. 1C,D), others (Dubal et al., 2022; Homem et al., 2014; Lee et al., 2016) have focused on the less-numerous ‘type II’ NSCs in the central brain (Fig. 1C,D) or have studied isolated NSCs in culture (Homem et al., 2014). Single-cell sequencing data indicate that metabolic differences exist between NSCs in different regions of the brain or at different developmental stages (Davie et al., 2018; Genovese et al., 2019). It will be interesting to compare how different stem cell identities, conditions and environments affect susceptibility to mitochondrial dysfunction. Not only within *Drosophila*, but also between model organisms. One major difference between *Drosophila* larval NSCs and their mammalian embryonic counterparts (or cells in culture), is the presence of a glial NSC niche in *Drosophila* (Fig. 1D). Glial cells share metabolites such as lactate and alanine with neurons, to fuel neuronal OxPhos activity (Magistretti and Allaman, 2018; Pellerin and Magistretti, 1994; Volkenhoff et al., 2015). It is conceivable that NSCs with access to glial support *in vivo* will also rewire their metabolic pathways based on the substrates on offer in the NSC niche, although this remains to be shown.

### OxPhos and glycolysis in mouse NSCs: cause or consequence?

To better understand the metabolic requirements of mammalian NSCs *in vivo*, mouse models have proven very useful, both during embryonic and adult mammalian neurogenesis (Fig. 1A,B). In the adult mouse brain (Mazumdar et al., 2010) or the rat carotid body (Bee et al., 1986), NSC proliferation occurs preferentially in hypoxic regions, suggesting that NSC self-renewal in these contexts occurs without mitochondrial respiration. During mouse and ferret brain development, the transition from symmetric to asymmetric divisions that marks the onset of neuronal differentiation coincides with vascularisation of the brain (Kuhnert et al., 2010; Lange et al., 2016; Lee et al., 2001). Preventing vascularisation favours NSC self-renewal at the expense of differentiation, which can be rescued by increased levels of systemic oxygen (Lange et al., 2016; Wagenführ et al., 2015). Interestingly, in *Drosophila*, symmetrically dividing NSCs of the optic lobe neuroepithelium are also more hypoxic than the asymmetrically dividing neurogenic NSCs in the central brain and ventral nerve cord (Baccino-Calace et al., 2020). Together, this may indicate a conserved difference in metabolic requirements between NSCs that undergo symmetric amplifying divisions versus asymmetric differentiating divisions.

Genetically inducing mitochondrial dysfunction in mouse embryonic NSCs has been achieved in different ways, leading to somewhat conflicting results. Early forebrain- (FoxG1-Cre) or cortex-specific (Emx1-Cre) deletion of the gene encoding the NADH-dependent mitochondrial oxidoreductase protein apoptosis inducing factor (AIF), results in microcephaly due to neuronal apoptosis and increased asymmetric divisions at the expense of NSC self-renewal (Khacho et al., 2017). In contrast, slightly later NSC-specific deletion (with hGFAP-Cre after E12.5) of either the complex II subunit SDHD (Díaz-Castro et al., 2015) or the complex I subunit NDUFS2 (Cabello-Rivera et al., 2019) causes only minimal changes to the brain at birth, apart from a subtle decrease in cortical and hippocampal thickness. These apparently conflicting data are probably related to different stages of recombination, different ways of perturbing mitochondrial function and selective analysis of the many transitions these NSCs undergo during brain development. Using the same hGFAP-Cre line, the impact of inhibiting complex I (Cabello-Rivera et al., 2019) on NSC proliferation *in vivo* seems to be more severe than that of complex II (Díaz-Castro et al., 2015). It will be interesting to see what this means for substrate use in embryonic NSCs and how each perturbation differentially affects electron transport, flux through the TCA cycle and metabolic rewiring.

Transcriptomic profiling of mouse NSCs at the onset of vascularisation and differentiation showed downregulation of glycolytic genes, without affecting OxPhos gene expression (Lange et al., 2016). A similar decrease in transcription of glycolysis, but not OxPhos, genes occurs during *in vitro* differentiation of human iPSC-derived NSCs (Zheng et al., 2016), accompanied by decreased glycolytic lactate production (Lange et al., 2016; Zheng et al., 2016). In these studies, OxPhos activity did not increase (De Bock et al., 2013; Lange et al., 2016) or only slightly increased (Zheng et al., 2016) between cultured NSCs and maturing neurons. This might suggest that it is different glycolytic activity that correlates with differentiation versus self-renewal of embryonic NSCs, rather than a switch between OxPhos and glycolysis. A key regulator of glycolytic gene expression, certainly in the context of hypoxia, is the hypoxia-inducible transcription factor HIF1a (Semenza, 2011). HIF1a is expressed and stabilised in embryonic and adult NSCs, and is required for NSC maintenance and induction of glycolysis gene transcription (Lange et al., 2016; Roitbak et al., 2011; Tomita et al., 2003). However, *in vitro* (Gustafsson et al., 2005) and in the adult mouse brain (Mazumdar et al., 2010), HIF1a also activates Notch and Wnt signalling, which promote NSC self-renewal. Therefore, it remains to be determined whether there is a direct effect of glycolysis on NSC proliferation, or whether high glycolytic activity is merely a consequence of HIF1a-activation in response to hypoxia. Interestingly, injecting the end-product of anaerobic glycolysis, lactate, in the neonatal mouse brain results in increased proliferation and subsequent NSC pool depletion (Álvarez et al., 2016), as does genetic inhibition of lactate efflux from the brain into the vasculature (Wang et al., 2019). These results may point to a direct impact of glycolysis, OxPhos and oxygenation on NSC behaviour, similar to what has been described in other developmental contexts (Miyazawa and Aulehla, 2018; Oginuma et al., 2020).

In the adult mouse brain, the situation is possibly more complicated because adult mouse NSCs are a heterogeneous population with large differences in division mode and neurogenic capacity. Numerous studies have shown adult NSCs to respond to hypoxia upon cerebral ischemia, mostly increasing neurogenesis in the different neurogenic regions of the adult mouse brain (reviewed by De Filippis and Delia, 2011; Li et al., 2021). Transcriptional analysis of the various stages of adult NSC differentiation has revealed downregulation of glycolysis genes and upregulation of complex V subunits (Box 1) specifically during activation from quiescence (Shin et al., 2015). Genetic manipulation of OxPhos activity does decrease NSC proliferation to some extent (Cabello-Rivera et al., 2019; Díaz-Castro et al., 2015; Khacho et al., 2017). However, these transcriptional changes do not necessarily translate into proteomic differences (Wani et al., 2022), and more-detailed analyses of metabolic requirements during specific NSC transitions have revealed a more complex picture. For example, deletion of the mitochondrial pyruvate carrier (MPC1), which is involved in the import of the end-product of glycolysis, pyruvate, into the mitochondria promotes both NSC activation from quiescence and differentiation into postmitotic neurons, but does not affect proliferation of activated NSCs itself (Petrelli et al., 2022 preprint). In contrast, long-term depletion of the mitochondrial transcription factor A (TFAM) mostly affects the self-renewal and survival of intermediate progenitor cells, a highly proliferative transient NSC stage shortly after reactivation (Beckervordersandforth et al., 2017). How this genetic long-term mitochondrial dysfunction affects cellular and niche metabolism, or many other aspects of adult NSC behaviour beyond proliferation, remains poorly understood.

## Mitochondrial morphology dynamics in NSCs

**Fig. 3.**
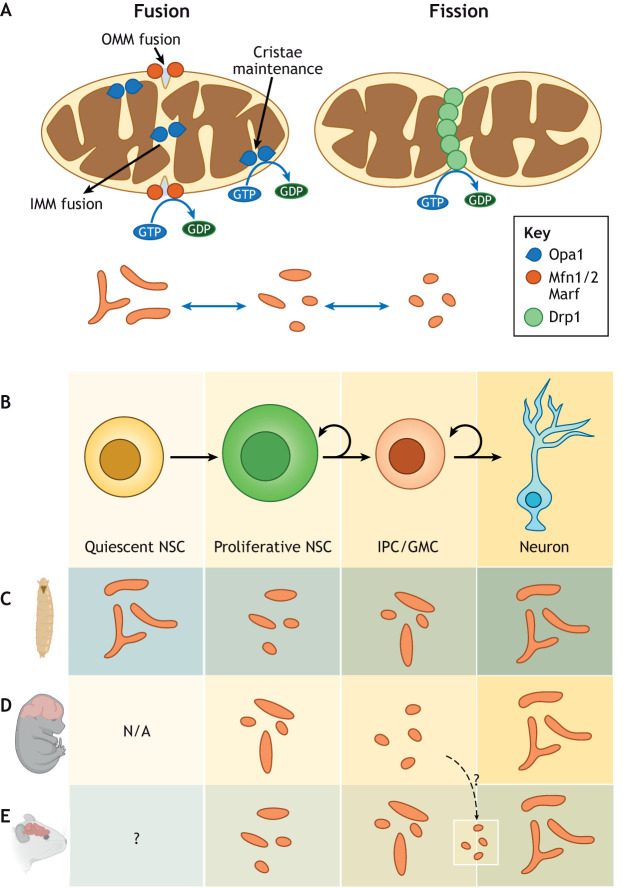
**Mitochondrial morphology during neurogenesis.** (A) Regulation of mitochondrial morphology by different GTPase proteins. Mitochondrial inner (IMM) and outer (OMM) membrane fusion is carried out by Opa1 and Mfn1 and/or Mfn2 (Mfn1/2) (Marf in *Drosophila*), respectively. Opa1 is also required for cristae maintenance. Mitochondrial fragmentation (fission) is regulated by Drp1. (B) Schematic representation of neurogenesis. (C) During *Drosophila* larval neurogenesis, quiescent neural stem cells (NSCs) exhibit fused mitochondrial morphology. When quiescent NSCs activate, mitochondria become fragmented, with some oblong mitochondrial particles. Intermediate neural progenitors (IPCs) and/or ganglion mother cell (GMCs) have intermediate morphology with fused and fragmented mitochondria. In mature neurons, mitochondria form highly fused structures. (D) Mouse embryonic proliferative NSCs have intermediate mitochondrial morphology. Mitochondria in IPCs are small and fragmented, while they exhibit fused morphology in differentiated neurons. (E) Proliferative NSCs in the dentate gyrus of the adult mouse brain show a mixture of globular and tubular mitochondrial morphologies, and are more elongated in proliferative IPCs. NSCs then mature into neurons where mitochondria have fused architecture. There is no quiescent stage in the mouse embryo (N/A, not applicable), and several processes remain undefined (question mark) in the adult mouse brain (see text).

In many cell types, mitochondria undergo morphological changes through cycles of fusion and fission, tightly regulated by the dynamin family of GTPases (Fig. 3A) (Tilokani et al., 2018). Mitochondrial fusion is regulated at the outer mitochondrial membrane by mitofusins (Mfn1 and Mfn2, Marf in *Drosophila*) and by Opa1 at the inner membrane. Mitochondrial fission is primarily regulated by Drp1. Additionally, Opa1 plays a role in the maintenance of cristae architecture along with OxPhos complex V (Cogliati et al., 2016; Patten et al., 2014). Mitochondrial morphology is intimately related to activity and function, and varies depending on cell type, stage of the cell cycle, ATP demand and other metabolic requirements of the cell (Kasahara et al., 2013; Mitra et al., 2009; Taguchi et al., 2007; Yao et al., 2019). In stem cells, such as haematopoietic and PSCs, mitochondria are functionally inactive and fragmented with poor cristae arrangement (Simsek et al., 2010; Xu et al., 2013; Zhang et al., 2011). In *Drosophila* larval NSCs, mitochondria are also considerably smaller and more fragmented than in neuronal progeny (Bonnay et al., 2020; Sen et al., 2013). However, rather than being fully fragmented, they are of intermediate morphology, containing a combination of punctate and somewhat oblong rod-like mitochondrial particles that are evenly distributed around the nucleus (Fig. 3B,C) and show clear activity (Lee et al., 2016; Van Den Ameele and Brand, 2019). Functional analysis of interactions between proteins regulating mitochondrial morphology and NSC behaviour has revealed that fusion is a key determinant of differentiation (Dubal et al., 2022) (Box 3). Loss of the pro-fusion proteins Opa1 or Marf in a subtype of *Drosophila* NSCs, type II neuroblasts, causes loss of neuronal progeny. However, depletion of Opa1 has a more severe effect on lineage size than Marf loss of function, indicating a possible role for Opa1-specific functions related to cristae architecture in NSC differentiation. Although depletion of the pro-fission protein Drp1 does not affect NSC proliferation, restoring fusion by co-depletion of Drp1 can rescue differentiation in Opa1 and Marf mutant NSCs (Dubal et al., 2022), highlighting the importance of mitochondrial fusion in NSC development.

**Box 3. Notch signalling and neural stem cell mitochondria**

Notch is one of the key signalling pathways required for neural stem cell (NSC) maintenance (Pierfelice et al., 2011). Activation of the pathway occurs when the extracellular domain of the Notch receptor (NECD) is bound by a ligand, resulting in cleavage of the intracellular domain (NICD) at the plasma membrane. The NICD translocates to the nucleus, where it associates with a DNA-binding protein [e.g. Suppressor of Hairless (Su(H)) in *Drosophila*] to activate Notch target genes. Previous studies have suggested a role for Notch in regulating glycolysis and oxidative phosphorylation (OxPhos) (de La Pena et al., 2001; Landor et al., 2011; Thorig et al., 1981), but the molecular details of how Notch and mitochondria influence each other remain unclear. In *Drosophila* type II NSCs (Fig. 1C,D), loss of Notch signalling causes mitochondrial fragmentation, while forced mitochondrial fusion rescues some aspects of Notch-dependent NSC proliferation and differentiation (Dubal et al., 2022). Notch-mediated NSC overproliferation is also rescued by inhibition of mitochondrial fusion, indicating a requirement for fused architecture to enable proper Notch signalling. Interestingly, Opa1 inhibition results in cytoplasmic accumulation of the NICD, possibly preventing it from entering the nucleus and activating target genes (Dubal et al., 2022). However, the precise intricacies of how Notch signalling, mitochondrial dynamics and cellular metabolism interact probably depend on the context of the cell or organism. When profiling chromatin occupancy of the NICD in NSCs of the mouse embryonic cortex, genes known to be involved in fusion and/or fission are not directly bound by the NICD (van den Ameele et al., 2022). Instead, in mouse embryonic neurospheres *in vitro*, mitochondrial fragmentation upon Opa1 knockdown causes a ROS-dependent increase in expression of the Notch-inhibitor Botch (also known as Chac1) (Khacho et al., 2016). In contrast, during cardiomyocyte development, similar mitochondrial fragmentation results in increased Notch activity, through increased Ca^2+^ and calcineurin signalling (Kasahara et al., 2013). Non-canonical transcription-independent functions of Notch may add to the complexity, as suggested for genetic interactions between Notch and mitophagy in *Drosophila* NSCs and brain tumours (Lee et al., 2013); or in HeLa cell culture, where non-canonical interactions between Notch and mitochondrial proteins prevent apoptosis (Perumalsamy et al., 2010). A mitochondrial targeting sequence in the NICD (Lee et al., 2011) leads to proteolytic cleavage by the protease mitochondrial intermediate peptidase (MIPEP) in HeLa cell culture. This mechanism may constitute a novel non-canonical pathway to regulate Notch activity. However, in contrast to other transmembrane receptors, such as the atypical cadherin Fat (Sing et al., 2014), mitochondrial localisation of Notch has, to the best of our knowledge, not yet been observed *in vivo*.

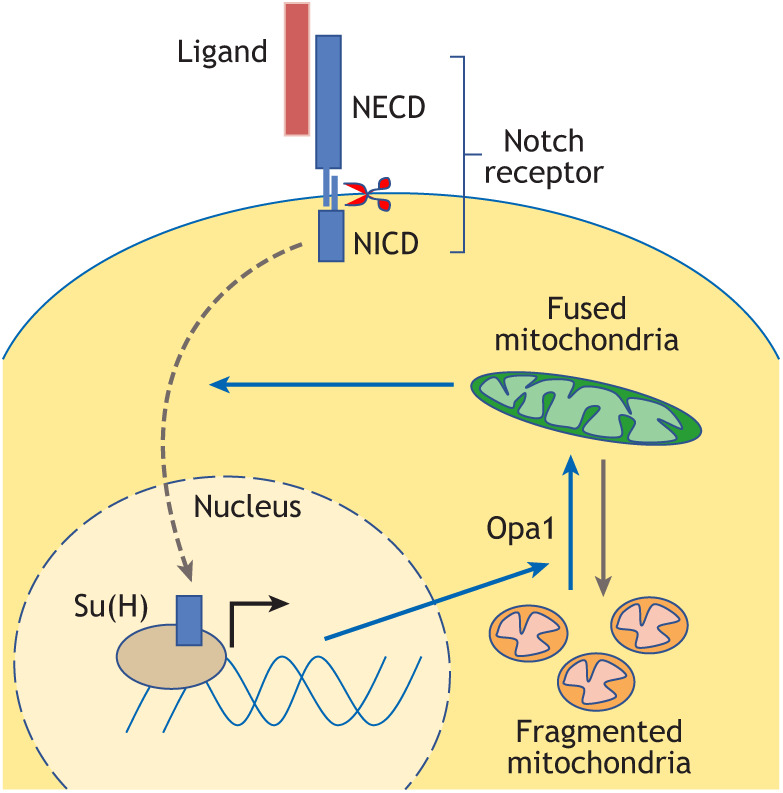

In contrast to *Drosophila*, proliferating NSCs in the embryonic mouse brain exhibit primarily fused mitochondria (Fig. 3D) (Iwata et al., 2020; Khacho et al., 2016). Fragmentation occurs during mitosis, as described in other contexts (Taguchi et al., 2007), but once mitosis is complete, the presumptive self-renewing daughter NSC reacquires a fused network, while the differentiating neuron or IPC initially maintains a fragmented morphology. Decreased fission through inhibition of Drp1 can promote NSC self-renewal at the expense of differentiation. Moreover, preventing fusion through depletion of Mfn1/2 or Opa1, causes a loss of NSC self-renewal and forces NSCs to commit to neuronal differentiation (Iwata et al., 2020; Khacho et al., 2016), indicating a causal relationship between mitochondrial morphology and NSC fate decision, as discussed below.

In the adult mouse brain, the interaction between mitochondrial morphology and NSC proliferation is less well understood. Postmitotic newborn neurons display a more elaborate and fused mitochondrial network than the activated hippocampal NSCs they derive from (Fig. 3E) (Beckervordersandforth et al., 2017). However, analysis of fission and fusion has mainly focused on neuronal maturation and apoptosis, e.g. in *Drp1* mutant mice (Ishihara et al., 2009; Steib et al., 2014; Wakabayashi et al., 2009). The few data on NSCs suggest that *Drp1* mutation and mitochondrial fusion decrease NSC proliferation in the postnatal cerebellum (Wakabayashi et al., 2009) and hippocampus (Steib et al., 2014). In contrast, decreased proliferation of hippocampal NSCs has also been observed upon aberrant mitochondrial fragmentation due to mutation of *Mfn1* and *Mfn2* (Khacho et al., 2016), suggesting roles of these different GTPases beyond mitochondrial fission and/or fusion, or an as yet incomplete understanding of the complex transitions these adult NSCs undergo. It is still unclear, for example, whether the mitochondria of *in vivo* quiescent NSCs are fragmented and immature, as observed *in vitro* (Cai et al., 2021; Petrelli et al., 2022 preprint), or whether they have a fused or clustered morphology akin to *Drosophila* (Fig. 3C,E) (Endow et al., 2019). It will also be interesting to see whether fission precedes neurogenesis from the various adult neural progenitor populations (Fig. 3E), similar to what is observed in the mouse embryo (Iwata et al., 2020).

Together, these findings indicate that changes in mitochondrial morphology are not a mere consequence of metabolic demand but may also act as upstream regulators in cell type specification. What the exact impact is on any given NSC fate transition depends on the cellular context, the organism and the environment they find themselves in. It is likely that the metabolic and molecular mechanisms that translate mitochondrial dynamics into cell fate decisions are equally diverse and context dependent. Given the many interactions between OxPhos and NSC behaviour, an obvious mechanism by which a change in mitochondrial architecture can affect NSC maintenance and differentiation is through its profound effect on OxPhos activity. Fused mitochondria are generally thought to be more efficient in ATP production due to efficient exchange of matrix metabolites (Westermann, 2012), dense cristae arrangement and ETC supercomplex formation (Cogliati et al., 2013, 2016). However, shifts in OxPhos activity also alter the NAD^+^/NADH redox balance, and many TCA cycle intermediates such as acetyl-CoA, succinate, fumarate or α-ketoglutarate that regulate activity of histone modifying enzymes (Lu and Thompson, 2012). The histone deacetylase Sirt1, a pro-neurogenic factor in the mouse embryonic brain (Bonnefont et al., 2019; Hisahara et al., 2008; Tiberi et al., 2012b), depends on a high NAD^+^/NADH ratio (Anderson et al., 2017). Therefore, increased fusion may inhibit neuronal differentiation by decreasing the NAD^+^/NADH ratio and inactivating Sirt1, as shown in mouse and human PSC-derived NSCs (Iwata et al., 2020). Another way to modulate cell fate is via second messenger molecules such as Ca^2+^, which is stored in mitochondria and the endoplasmic reticulum. Mitochondrial Ca^2+^ buffering regulated by phosphorylation of Miro at the endoplasmic reticulum contact sites regulates *Drosophila* NSC maintenance and lineage progression (Lee et al., 2016). In humans, ARHGAP11B, a human-specific gene involved in expansion of the cerebral cortex (Florio et al., 2015), is thought to cause expansion of basal NSCs by increasing their mitochondrial Ca^2+^ content and promoting glutaminolysis, through direct interaction with IMM proteins (Namba et al., 2020). However, it is unclear how elevated levels of mitochondrial Ca^2+^ relate to the changes in mitochondrial morphology that also accompany these human NSC transitions (Iwata et al., 2020). A change in Ca^2+^ is likely to impact the processing and activity of key signalling pathway factors either activating or inhibiting the pathway. For example, increased cytoplasmic Ca^2+^ due to lack of buffering capacity by fragmented mitochondria in Opa1- and Mfn1-depleted cardiomyocytes increases calcineurin activation and Notch processing, thereby activating the Notch signalling pathway (Kasahara et al., 2013) (Box 3). Upon differentiation, neuronal axon branching is also regulated by mitochondrial fission to decrease its Ca^2+^ buffering capacity and neurotransmitter release potential (Lewis et al., 2018). Conversely, in long-term potentiation, changes in intracellular Ca^2+^ can cause an increase in mitochondrial fission due to activation of CAMKII and Drp1 phosphorylation (Divakaruni et al., 2018). Many other mechanisms may communicate changes in mitochondrial morphology to instruct cell behaviour. Further analysis of mitochondrial cristae density and structure (Teixeira et al., 2015), mitochondrial membrane potential (Tomer et al., 2018), ETC activity, levels of different metabolites or pH (Oginuma et al., 2020), and their impact on nuclear chromatin and signalling pathways in NSC development will shed further light on the intricate relationship between neurogenesis and mitochondrial dynamics.

## Conclusions

When observing a small NSC in a dish, seeing it divide and then watching it mature into a large, elaborate, highly connected and electrically active neuron (Gaspard et al., 2008), it is easy to imagine that each transition must be accompanied by significant rewiring of metabolic pathways to fuel DNA replication, membrane growth or action potentials. We are only now starting to understand the basic differences in metabolic requirements of proliferation versus differentiation (Vander Heiden et al., 2011). Much remains to be discovered about how metabolism not only supports but also fuels the many other transitions that NSCs undergo, in different niches and different organisms, exposed to different and highly variable environments. Many of the current bulk metabolite or flux analyses do not account for the heterogeneity between various NSCs, even within the same organism, as suggested from single-cell transcriptomics (Genovese et al., 2019). Use of genetically encoded sensors *in vivo* (Gándara et al., 2019; Hudry et al., 2019; Tsuyama et al., 2013) or the steadily increasing resolution of spatial metabolomics techniques (Rappez et al., 2021) provide promising prospects (Mosteiro et al., 2021). These may also provide insight in the metabolic interactions between NSCs and their niche. Exchange of metabolites between different cell types is well established, e.g. in tumours (Lau and Vander Heiden, 2020) or between neurons and astrocytes (Magistretti and Allaman, 2018; Volkenhoff et al., 2015). It will be interesting to see whether presence or absence of glial support cells during the various stages of brain and NSC development (Fig. 1D) may dictate dependence on OxPhos or specific nutrients and metabolic pathways.

Many other aspects of mitochondria in development and disease, beyond their morphology, quality control, ROS or ATP production remain to be explored in NSCs. Intriguing observations of mitochondria being trafficked between different cell types (Boukelmoune et al., 2018; Peruzzotti-Jametti et al., 2021), acting as hubs for key signalling pathways (Sing et al., 2014), segregating and suffering from mtDNA mutations (Klein Gunnewiek et al., 2020; Lorenz et al., 2017; Ross et al., 2013; Van Den Ameele et al., 2020; Wang et al., 2010, 2011) or showing cristae maturation independent of OxPhos activity (Teixeira et al., 2015) are all worth pursuing. In addition, mitochondria play an important role in lipid droplet homeostasis and fatty acid β-oxidation, the impacts of which on embryonic and adult NSC behaviour have been studied extensively (Bailey et al., 2015; Hamilton et al., 2015; Knobloch et al., 2013, 2017; Xie et al., 2016), but the effects of which on NSC behaviour and signalling pathways probably reach beyond simply providing substrates for energy or building blocks (Dong et al., 2021) (Box 2).

Finally, it is worth noting that primary mitochondrial disorders, caused by nuclear or mitochondrial encoded mutations in OxPhos genes, are rarely accompanied by obvious microcephaly (Falk, 2010), apart from some interesting exceptions (Baum and Gama, 2021; Iwata and Vanderhaeghen, 2021). From the previous paragraphs, it has become clear that interactions between metabolism, development and disease are highly context dependent, and differences between humans and model systems, both *in vitro* and *in vivo*, remain to be accounted for.
